# Tricyclic Benzo[*cd*]azulenes Selectively Inhibit Activities of Pim Kinases and Restrict Growth of Epstein-Barr Virus-Transformed Cells

**DOI:** 10.1371/journal.pone.0055409

**Published:** 2013-02-06

**Authors:** Alexandros Kiriazis, Riitta L. Vahakoski, Niina M. Santio, Ralica Arnaudova, Sini K. Eerola, Eeva-Marja Rainio, Ingo B. Aumüller, Jari Yli-Kauhaluoma, Päivi J. Koskinen

**Affiliations:** 1 Division of Pharmaceutical Chemistry, Faculty of Pharmacy, University of Helsinki, Finland; 2 Department of Biology, University of Turku, Finland; 3 Pharmacy Section, FinPharma Doctoral Program, Finland; 4 Drug Discovery Section, FinPharma Doctoral Program, Finland; University of Texas M.D. Anderson Cancer Center, United States of America

## Abstract

Oncogenic Pim family kinases are often overexpressed in human hematopoietic malignancies as well as in solid tumours. These kinases contribute to tumorigenesis by promoting cell survival and by enhancing resistance against chemotherapy and radiation therapy. Furthermore, we have recently shown that they increase the metastatic potential of adherent cancer cells. Here we describe identification of tricyclic benzo[*cd*]azulenes and their derivatives as effective and selective inhibitors of Pim kinases. These compounds inhibit Pim autophosphorylation and abrogate the anti-apoptotic effects of Pim kinases. They also reduce cancer cell motility and suppress proliferation of lymphoblastoid cell lines infected and immortalized by the Epstein-Barr virus. Thus, these novel Pim-selective inhibitors provide promising compounds for both research and therapeutic purposes.

## Introduction

Recently there has been enormous progress in developing small molecule inhibitors against different types of protein kinases, including multiple compounds targeting Pim kinases [1], [2], [3]. The three Pim family members (Pim-1, Pim-2 and Pim-3) form an evolutionary distinct subgroup of serine/threonine-specific kinases that structurally belong to the group of calcium/calmodulin-dependent protein kinases. Pim kinases are highly homologous to each other and have partially overlapping functions and expression patterns [4], [5]. Unlike most other kinases, Pim kinases are constitutively kept in an active conformation [6], which is why their activities correlate well with their expression levels. In hematopoietic cells, expression of Pim kinases is upregulated by numerous growth factors and cytokines such as interleukins [7], [8], [9]. When overexpressed, Pim kinases are oncogenic and have been implicated both in hematopoietic malignancies such as leukemias and lymphomas [10] and in solid tumors such as prostate, colon, oral, hepatic and pancreatic cancers [11], [12].

Pim kinases promote tumorigenesis by supporting cell survival [13] and by enhancing resistance of cancer cells against chemotherapy [14] and radiation therapy [15]. At molecular level, Pim kinases regulate activities of several cellular transcription factors such as c-Myb [16], NFATc1 [17], STAT5 [18] and the RUNX family proteins [19]. Also viral factors are affected, including the Epstein-Barr virus (EBV) nuclear antigen EBNA2 [20] and the Kaposís sarcoma-associated herpesvirus (KSHV) latency-associated nuclear antigen LANA [21]. In addition, all Pim family members phosphorylate and thereby inactivate the pro-apoptotic Bad protein [22], [23], [24]. All these data may explain why Pim kinases so efficiently cooperate with Myc family transcription factors in development of lymphoid or solid tumors. Even though Myc-overexpressing cells proliferate faster, they are more prone to apoptosis, so a growth advantage is given to cells co-overexpressing also the anti-apoptotic Pim kinases. These conclusions are supported by recent reports [25], [26] showing that Pim-1 synergizes with Myc both to induce advanced prostate carcinoma and to maintain tumorigenicity of the cancer cells. Furthermore, we have recently demonstrated that Pim kinases increase the metastatic potential of adherent cancer cells [27]. For all these reasons, Pim kinases have become increasingly attractive targets for cancer therapy. Furthermore, mice lacking activities of all the three Pim family members show only a fairly mild phenotype with slightly reduced growth responses [5], so compounds selectively inhibiting Pim activity are not expected to have severe adverse side effects.

Here we have analysed the biological effects of new tricyclic benzo[*cd*]azulenes, the synthesis methodology of some of which we have recently described [28], [29], [30]. We have identified several of them as effective and selective inhibitors against Pim family kinases, and demonstrate that these inhibitors can efficiently interfere with both biochemical and intracellular activities of Pim kinases. We have also found evidence that these compounds or their derivatives could be useful in development of therapies to treat metastatic tumors and/or to prevent of EBV-induced lymphomagenesis.

## Results

### Synthesis of Tricyclic Benzo[*cd*]azulenes and their Novel Derivatives

**Figure 1:**
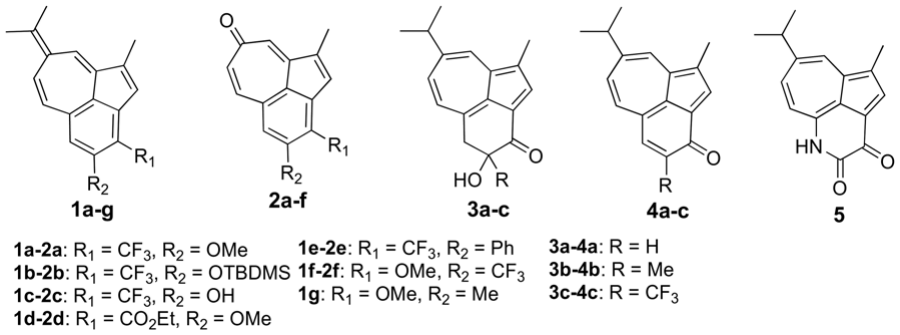
Chemical structures of some new and previously synthesized benzo[*cd*]azulenes.

**Figure 2:**
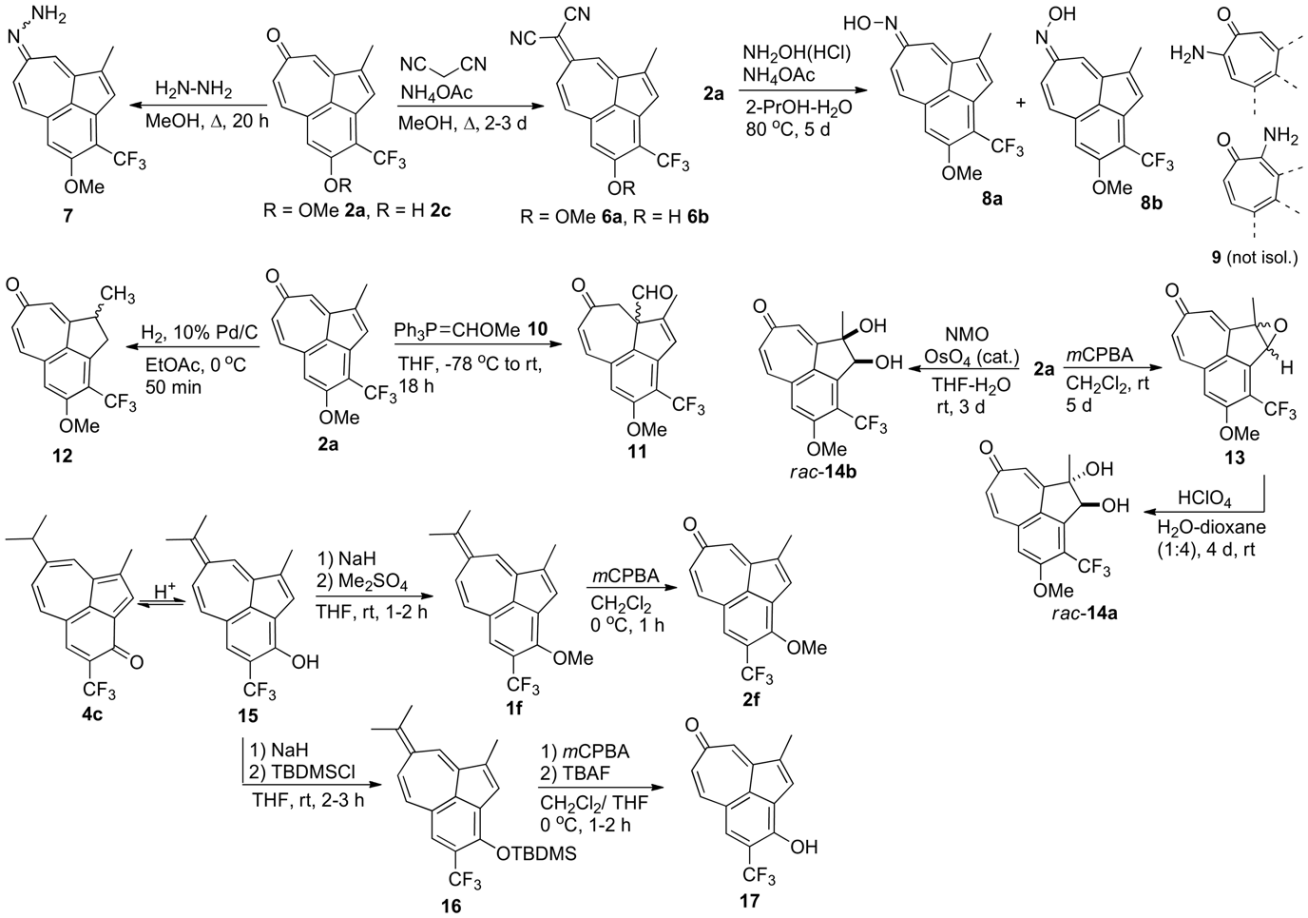
Synthetic steps in the preparation of new benzo[*cd*]azulenes.

We have reported a facile one-pot method for transformation of guaiazulene derivatives into tricyclic heptafulvenes **1a–e** [28]. The starting azulenes were treated with an appropriate base to furnish the new tricyclic benzo[*cd*]azulene skeleton with a functionalized, fused six-membered ring (Figure **1**). Furthermore, we discovered that the heptafulvenes **1a–e** were prone to oxidative cleavage when treated with a mild oxidant *m*CPBA (*meta*-chloroperoxybenzoic acid), yielding the corresponding tricyclic tropones **2a–e** (Figure **1**) [28]. In addition, we have recently described the facile synthesis of tricyclic benzo[*cd*]azulen-3-ones **3a–c**, **4a–c** and **5** [29], [30] from commercially available guaiazulene. There we had used acid-catalyzed tautomerization reactions to further transform benzo[*cd*]azulen-3-ones **4b–c** to regiosiomeric heptafulvenes **1f–g**. When **1f** carrying the trifluoromethyl substituent at 4-position was now modified by oxidation (see Figure 2 below), we obtained a novel tropone **2f** as a regioisomer of **2a**.

### Benzo[*cd*]azulenes are Selective Inhibitors against Pim Family Kinases

Six of the originally synthesized compounds were tested at 10 µM concentration against a panel of 71 different kinases together with their optimized peptide substrates, as previously described [31] and screened for residual kinase activities less than 50%. Interestingly, as shown in Table **1**, two of the compounds (**1a** and **4c**) significantly reduced the *in vitro* activities of Pim family members, especially those of Pim-1 and Pim-3. **1a** was clearly more selective than **4c**, but showed some inhibitory activity also against PRAK, p38g and some DYRK family members. Also **2a** and **2f** were fairly active against Pim-1 and Pim-3 and were even more selective than **1a**, but **2f** also efficiently targeted EF2K. By contrast, **1e** and **4b** showed hardly any activity in any of the *in vitro* kinase assays. Here it should be noted that a screen like this gives only preliminary estimates on kinase specificity of the compounds, which is why the results need to be validated by other means.

**Table 1:** Selectivity of benzo[*cd*]azulenes against recombinant kinases.

Kinase	Residual activity (%)					
Compound	1a	1e	2a	2f	4b	4c
ERK1	114	114	126	111	121	86
ERK2	109	91	104	94	109	108
JNK1	97	84	86	96	109	73
JNK2	89	87	86	95	104	83
p38a MAPK	98	94	91	100	123	95
P38b MAPK	93	93	96	102	110	99
p38g MAPK	62	117	90	75	106	113
p38s MAPK	90	105	98	84	93	95
ERK8	94	103	101	83	101	55
GSK3b	128	97	97	112	105	99
CDK2-Cyclin A	85	96	87	91	78	87
DYRK1A	56	92	82	97	107	91
DYRK2	99	80	104	84	88	67
DYRK3	63	90	90	90	89	55
SRPK1	90	99	92	100	113	76
HIPK2	76	83	68	83	87	52
RSK1	92	104	87	104	100	91
RSK2	100	76	110	89	106	64
PDK1	68	101	105	93	69	113
PKBa	90	97	102	101	115	92
PKBb	150	78	83	161	157	54
SGK1	103	105	101	78	109	81
S6K1	92	90	73	96	115	77
PKA	129	106	97	102	98	107
ROCK 2	94	96	95	97	100	93
PRK2	100	106	100	92	111	116
PKCa	72	86	72	99	101	80
PKC zeta	107	98	97	87	100	66
MSK1	108	108	101	102	109	79
PKD1	107	93	103	94	95	97
MNK1	96	101	96	77	103	103
MNK2	67	74	78	65	86	80
MAPKAP-K2	81	87	80	83	100	58
PRAK*	56	69	72	89	93	42
CAMK1	78	76	102	97	100	75
SmMLCK	73	92	74	78	89	90
PHK	94	99	118	100	105	100
CHK1	128	98	87	95	101	128
CHK2*	88	90	90	106	114	35
AMPK	95	101	94	96	97	99
MARK3	110	100	93	99	111	105
BRSK2	69	196	294	85	80	126
MELK	90	102	106	102	98	63
**PIM-1** *	**42**	**86**	**63**	**58**	**84**	**50**
PIM-2	72	110	103	95	95	83
**PIM-3** *	**30**	**80**	**56**	**66**	**82**	**24**
CK1	108	114	116	114	108	101
CK2	90	101	94	103	107	92
MKK1	81	77	70	85	88	78
MST2	109	97	114	114	108	94
PAK4	82	90	101	92	86	83
PAK5	82	82	77	90	99	80
PAK6	110	101	109	96	111	99
MST4	95	104	85	99	77	69
Src	93	90	99	104	104	102
Lck	91	89	79	88	94	77
CSK	94	79	78	98	97	75
FGF-R1	100	105	110	106	100	51
IRR	78	93	87	74	94	84
EPH A2	105	100	99	96	90	96
SYK	111	123	132	128	118	65
YES1	116	99	91	82	96	54
EF2K*	73	137	116	39	62	129
CAMKKb	100	103	98	105	95	108
NEK2a	98	118	131	96	99	69
NEK6	122	105	141	101	110	73
IKKb	103	112	105	108	96	94
PLK1	99	93	117	93	100	60
PLK1 (okadaic acid)	88	80	71	82	82	59
IKKe	85	85	83	105	105	59
TBK1	98	92	106	104	103	99

Kinase assays were carried out at 10 µM concentrations of benzo[*cd*]azulenes dissolved in DMSO. Residual activity of the kinases is shown.

* marks for residual activity ≤50%.

### Benzo[*cd*]azulenes Abrogate Anti-apoptotic Effects of Pim-1 in Cytokine-deprived Myeloid Cells

To determine whether benzo[*cd*]azulenes can enter the cells and inhibit intracellular Pim kinase activity, we carried out cell-based assays with FDCP1 murine myeloid cells that are strictly dependent on the cytokine interleukin-3 (IL-3) for their growth and survival. In these assays, we used previously characterized FDCP1-derived cell lines that had been stably transfected with either neomycin (FD/Neo) or the 44 kD isoform of Pim-1 (FD/Pim44) [13]. Since continuous Pim-1 activity significantly prolongs survival of FDCP1 cells in the absence of IL-3 [23] it was anticipated that after IL-3 withdrawal, an effective Pim inhibitor would reduce the survival of FD/Pim44 cells to the level of FD/Neo cells, but would not have severe cytotoxic effects. To quantitate the effects of the test compounds on cell viability, we either measured the metabolic activity of the cells with the MTT assay or counted Trypan blue-excluding live cells.

**Figure 3:**
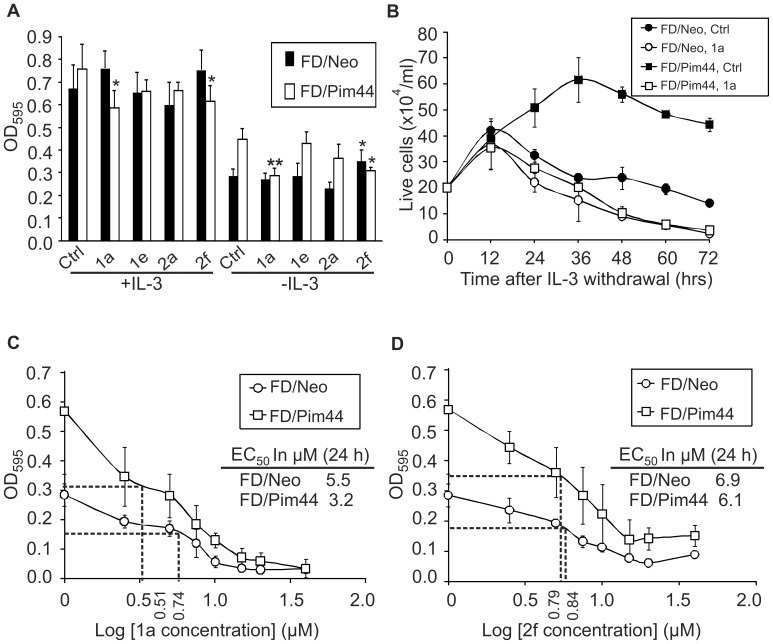
Benzo[*cd*]azulenes inhibit Pim-1-dependent cell survival. (**A**) FDCP1 cell lines stably expressing neomycin (FD/Neo) or Pim-1 (FD/Pim44) were cultured for 24 h with or without IL-3 in the presence of DMSO or 5 µM inhibitors, after which cell viability was analyzed by the MTT assay. Graph represents means and standard deviations from two independent experiments with duplicate samples. Statistically significant differences between inhibitor-treated cells as compared to DMSO-treated control cells have been marked with asterisks. (**B**) Cells grown in the absence of IL-3 were stained with Trypan blue and live cells were counted at the indicated time-points. Points represent means and standard deviations from triplicate determinations from one of two similar experiments. (**C–D**) Cells were cultured for 24 h without IL-3 in the presence of increasing concentrations of either **1a** (**C**) or **2f** (**D**). Cell viability was analysed by the MTT assay and EC_50_ values were determined. Points represent means and standard deviations from three independent experiments with duplicate samples.

When FDCP1 derivatives were cultured for 24 h in the presence of serum and DMSO, but in the absence of IL-3, FD/Pim44 cells remained metabolically more active than FD/Neo cells (Fig. 3A, Ctrl, right panel), as was expected based on our previous results [23], [27]. In the presence of IL-3, there was no such difference between these two types of cells (Fig. 3A, Ctrl, left panel). However, when IL-3-deprived cells were treated with 5 µM test compounds dissolved in DMSO, **1a** and **2f** reduced the metabolic activity of FD/Pim44 cells significantly to the level of FD/Neo cells (Fig. 3A, right panel). In the presence of IL-3, **1a** and **2f** did not display any general cytotoxicity on FD/Neo cells, but slightly reduced the metabolic activity of FD/Pim-cells (Fig. 3A, left panel), suggesting that this stable cell line has become addicted to continuous expression of Pim-1. By contrast, compounds **2a**, **1e** and **4b** remained repeatedly ineffective in all these assays, while **4c** displayed strong non-specific cytotoxicity in both types of cells (Fig. 3A and data not shown). Altogether, these results indicated that some, but not all of the tested benzo[*cd*]azulenes are effective cell-permeable Pim kinase inhibitors that can siginificantly impair the anti-apoptotic effects of Pim-1.

To measure cell viability more directly, we stained cells with Trypan blue and counted dye-excluding live cells at multiple time-points after withdrawal of IL-3. As shown in Fig. 3B, FD/Neo cells treated with DMSO ceased to proliferate and started to die already after 12 h, while FD/Pim44 cells continued their growth much longer and were still mostly alive after 72 h. However, when cells were treated with 5 µM **1a** or **2f**, the protective effects of Pim-1 were completely lost and both types of cells died within 72 h (Fig. 3B and data not shown). These results indicated that results from the MTT assay on metabolic activity of cells reliably reflect also cell viability.

To determine the effective concentrations (EC_50_) of **1a** and **2f** that would reduce the viability of cells by 50%, IL-3-deprived FD/Neo and FD/Pim44 cells were cultured with increasing doses of the inhibitors and analysed 24 h later by the MTT assay. Calculation of the EC_50_ values for **1a** or **2f** indicated that they were nearly similar in both FD/Neo (5.2 and 6.9 µM) and FD/Pim44 (3.2 and 6.1 µM) cell lines, respectively (Fig. 3C–D).

### Benzo[*cd*]azulenes Prevent Cancer Cell Migration

**Figure 4:**
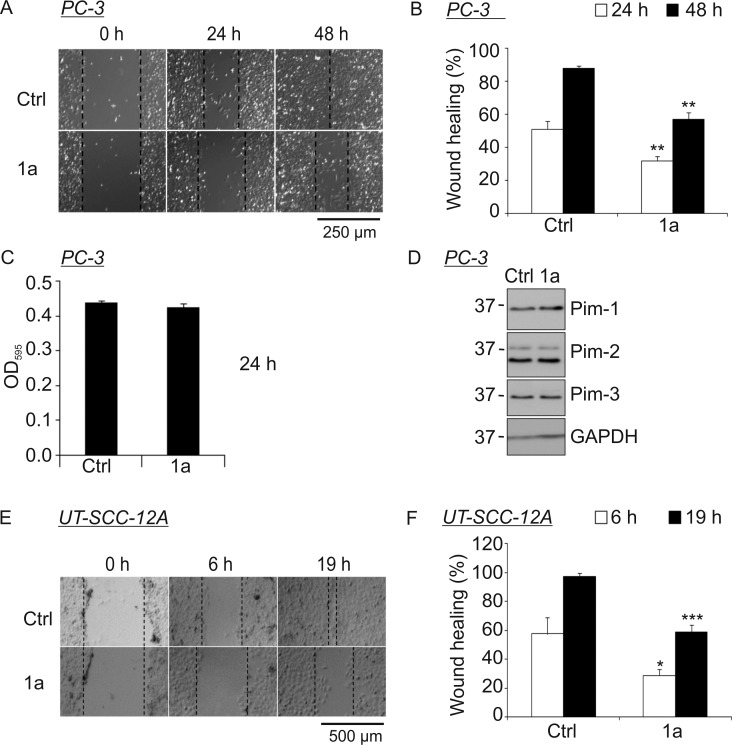
Benzo[*cd*]azulene 1a decreases cancer cell migration without affecting cell viability or Pim protein levels. (**A–B**) PC-3 cells were cultured on 24-well plates, treated with either 0.1% DMSO (Ctrl) or 10 µM **1a** and scratched with a sterile 200 µL pipette tip. Pictures were taken at indicated time-points and analyzed. Shown are representative pictures from each time-point. The graph represents means and standard deviations from triplicate samples. (**C**) MTT assay was used to study the effects of DMSO and **1a** on PC-3 cell viability. Shown are means and standard deviations from duplicate samples from one of two similar experiments. (**D**) Western blotting with antibodies specific for each Pim family member was carried out with samples of PC-3 cells that had been treated for 24 h with either 0.1% DMSO (Ctrl) or 10 µM **1a**. Shown is a representative picture from two independent experiments. (**E–F**) Wound healing assays were performed with the UT-SCC-12A cell line similarly to the PC-3 cell line. Shown are representative pictures from indicated time-points. The graph represents means and standard deviations from triplicate samples.

We have recently shown that Pim family kinases enhance motility of adherent cancer cells [27]. Therefore, we wanted to analyse the ability of **1a** to prevent migration of PC-3 prostate cancer cells as well as UT-SCC-12A head and neck squamocellular carcinoma cells. For this purpose, confluent cells were treated with either DMSO or 10 µM **1a**. Two hours later, wounds were scratched across the cell monolayer and pictures were taken at distinct time-points to follow up the healing process. Results from these scratch wound assays clearly indicated that **1a** decreases the motility of both PC-3 cells (Fig. 4A–B, Movies S1, S2) and UT-SCC-12A cells (Fig. 4E–F). By contrast, metabolic activities of both types of cells and cellular expression of Pim family members were not significantly affected by **1a**, as measured by the MTT assay (Fig. 4C and data not shown) and by Western blotting (Fig. 4D and data not shown), respectively. Furthermore, these results were well in line with those previously obtained using the Pim-selective inhibitor **DHPCC-9** or Pim-specific RNA interference reagents [27], suggesting that the effects of also benzo[*cd*]azulenes are specifically targeted against intracellular Pim kinase activity.

### Benzo[*cd*]azulenes Prevent Proliferation of Lymphoblastoid Cell Lines

**Figure 5:**
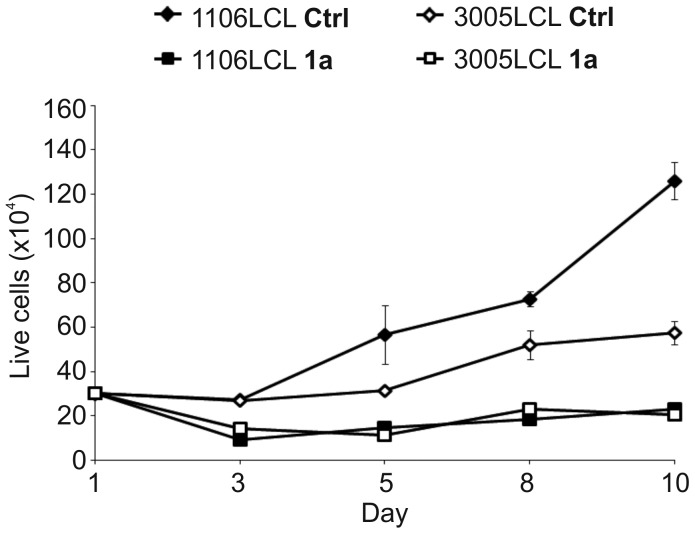
Benzo[*cd*]azulenes prevent proliferation of lymphoblastoid cell lines. LCL cell lines were cultured for up to 9 days in optimal density on 6-well plates and treated with either 0.1% DMSO (Ctrl) or 10 µM **1a**. Viable cells were counted by Trypan blue staining. Shown are means and standard deviations from two parallel samples in one representative experiment.

We have previously demonstrated that Epstein-Barr virus (EBV) upregulates expression and activity of Pim family proteins in the hosting B-cells and that Pim kinases in turn stimulate the transactivation activity of the EBV nuclear antigen 2 (EBNA2) [20]. To determine whether maintenance of high Pim activity is essential for proliferation of EBV-infected and immortalized lymphoblastoid cell lines (LCLs), we picked up two such cell lines, and propagated them in the presence of DMSO or 10 µM **1a**. Cells were grown for up to 9 days and kept in an optimal density by adding more medium together with either DMSO or **1a**. When live cells excluding Trypan blue were counted on alternate days, the DMSO-treated control cells steadily continued their proliferation; while the cells treated with **1a** slowed down or even completely stopped growth, but did not die out, either (Fig. 5). Similar effects by **1a** were obtained, whether the medium was completely replaced during the experiment or whether conditioned medium was used (data not shown).

### Synthesis of Additional Benzo[*cd*]azulene Derivatives to Identify more Effective Pim Inhibitors

Based on the promising results on Pim kinase inhibition in multiple cell-based assays, we embarked on further modification of the azulenes, heptafulvenes and tropones presented in Figure **1**. The aim was to develop derivatives that would be even more potent as Pim kinase inhibitors than the original benzo[*cd*]azulene compounds.

Tropone **2a** [28] was used as a key intermediate, since it was easily accessible in high yield (82%) from the parent heptafulvene **1a** that was shown to be an effective and selective Pim kinase inhibitor. In addition, the tropones were generally found to be chemically more stable than the corresponding heptafulvenes. The exocyclic double bond was restored in a single transformation, when **2a** was subjected to the Knoevenagel condensation with malononitrile in a reaction catalysed by ammonium acetate to give highly conjugated dicyanoheptafulvene **6a** (analogous two-step synthesis *via* ethoxytropylium fluoroborate [32]) in 45% yield (MeOH, reflux, 2–3 d, Figure 2). This crystalline product has a good chemical stability in aqueous solutions. Since demethylation of the methoxy group on tropones **2a** and **2f** under standard conditions (BBr_3_, 2–4 equiv., CH_2_Cl_2_, rt, 2–8 h) was found to be unsuccessful, the free phenol analogue **2c** (Figure **1**) [27] was synthesized and subjected to the Knoevenagel condensation (malononitrile, MeOH, reflux, 4 d) to give the phenolic dinitrile **6b** (Figure 2). In the presence of hydrazine monohydrate (MeOH, reflux, 20 h), the carbonyl group of **2a** was transformed into hydrazide product **7** (Figure 2), which was isolated as an inseparable mixture of two diastereomers (*Z*/*E*, ^1^H NMR).

The carbonyl group of **2a** was transformed into the oxime functionality by treating it with hydroxylamine hydrochloride in the presence of base in a mixture of isopropanol–water (3∶1) for a prolonged time (80°C, 5 d, Figure 2). Two stereoisomers were separated by a careful column chromatography on silica gel followed by recrystallization from ethyl acetate/*n*–hexane to give pure *Z* and *E* isomers (NMR, NOE assignment) of oximes **8a** (29%, orange needles) and **8b** (42%, yellow needles). No 2-aminotropone derivatives **9** were isolated as reported previously for the tropone itself to produce a mixture of products under the same reaction conditions [33].

In the presence of phosphonium ylides the α,ß-unsaturated ketone moiety of tropone **2a** was found to undergo 1,4-conjugate addition reaction instead of the expected Wittig reaction. A related reaction type has been reported previously [34], [35]. The ylide **10** [36] was allowed to react with **2a** at low temperature (–78°C) to give one main product **11** in 38% yield after aqueous acidic work-up and chromatographic purification. Extensive 2D NMR (HMBC, HSQC, and NOESY) analysis revealed that **11** had an unexpected structure of a quaternary aldehyde with a non-planar junction between the fused seven and five-membered rings (Figure 2).

Catalytic hydrogenation of **2a** gave one main product after chromatographic isolation. Instead of reduction of the double bond in the seven member ring system reported for 3,4-fused benztropone [33], it was found that the double bond in the 5-membered ring of **2a** was highly susceptible to catalytic hydrogenation, when the reaction conditions were carefully controlled (Figure 2, H_2_, 10% Pd/C, EtOAc, 0°C, 50 min). The racemic non-planar compound **12** was obtained in 40% yield. The C = C double-bond in a five-member ring showed regioselectivity towards oxidation, when tropone **2a** was treated with excess of *m*CPBA for prolonged time (3 eq, rt, 5 d) giving the racemic epoxide **13** as a main product in 42% yield after column chromatography. The oxirane ring in **13** was prone to acid-catalyzed ring opening (perchloric acid) to give *trans*-diol **14a** in high yield (89% after column chromatography). The corresponding *cis*-diol **14b** was synthesized directly from the alkene **2a** by using catalytic amount of osmium tetroxide (OsO_4_) and *N*-methylmorpholine *N*-oxide (NMO) as a co-oxidant. Both **14a** and **14b** showed improved solubility in protic solvents and were colourless as compared to the previously synthesized strongly coloured tropones (yellow/orange).

We had recently reported a tautomerization reaction that proceeds *via* isomerization of π-bonds across the azulene moieties of tricyclic benzo[*cd*]azulen-3-ones **4a–c** synthesized from the parent 4,5-dihydrobenzo[*cd*]azulen-3-ones **3a–c** [29]. This efficient synthetic route had produced heptafulvenes **1f** and **1g** (Fig. **1**) with the regioisomeric substitution pattern on the aromatic six-membered ring. Using *m*CPBA in CH_2_Cl_2_ at 0°C, we were now able to oxidize the heptafulvene derivative **1f** [29] to obtain the novel tropone **2f** in high yield (85%) (Figure **1**, Figure 2). By contrast, isolation of the phenol tautomer **15** of benzo[*cd*]azulen-3-one **4c** was not possible due to reverse nature of the tautomerization reaction, so phenolic products could not be synthesized. Demethylation of the aryl methyl ethers on tropones **2a** and **2f** to phenolic products with standard reagent (BBr_3_) was also inefficient. However, to overcome this problem, a cleavable phenolic tautomer could be trapped as a silyl enol ether. This strategy was demonstrated in the efficient two-step synthesis, where the phenol tautomer was generated first *in situ* (HCl, cat., THF, rt, 20 min) and, after deprotonation, derivatized by silylation (NaH, 5 equiv and TBDMSCl 2.5 equiv, rt, 2–3 h) to give **16** in high 84% yield (Figure 2). This allowed the *m*CPBA oxidation of the *exo*-double bond of heptafulvene **16**, followed by a removal of the TBDMS-protection group by a 1.0 M solution of TBAF (tetrabutylammonium fluoride) in THF (1.2 equiv, THF, rt, 2 h) to finally give the troponoyl phenol **17** (Figure **1**, Figure 2) as a regioisomer of the benzo[*cd*]azulene **2c**.

### Some Benzo[*cd*]azulene Derivatives are Effective as Pim Inhibitors

To determine the efficacy of previously or newly synthesized compounds, we performed *in vitro* kinase assays with bacterially produced human Pim-1 protein and measured its residual activity in the presence of 10 µM concentrations of the compounds. The previously tested compounds **1a**, **1e**, **2a**, **2f**, **4b** and **4c** were used as positive controls to succesfully confirm that the newly obtained results shown in Table **2** were within the same range as those shown in Table **1**. By contrast, the other benzo[*cd*]azulenes and their derivatives tested inhibited the autophosphorylation activity of Pim-1 to a variable extent. Several of them were as effective as the parental ones, but some were even more potent. The most striking results were obtained with **2c**, which repeatedly reduced the autophosphorylation activity of Pim-1 by up to 89%.

**Table 2:** Efficacy of benzo[*cd*]azulenes as Pim-1 inhibitors *in vitro* and in cell-based assays.

Structure	Compound code, reference	Residual Pim-1activity *in vitro*	Residual viability ofFD/Neo cells	Residual viability ofFD/Pim44 cells
<$>\raster="rg6"<$>	**1a** 1	43	94	65
<$>\raster="rg7"<$>	**1c** 1	29	N.D.	N.D.
<$>\raster="rg8"<$>	**1e** 1	59	100	96
<$>\raster="rg9"<$>	**1f** 2	46	74	97
<$>\raster="rg10"<$>	**1g** 2	105	N.D.	N.D.
<$>\raster="rg11"<$>	**6a**	60	78	61
<$>\raster="rg12"<$>	**6b**	41	87	93
<$>\raster="rg13"<$>	**2a** 1	74	80	81
<$>\raster="rg14"<$>	**2c** 1	11	85	83
<$>\raster="rg15"<$>	**2d** 1	98	N.D.	N.D.
<$>\raster="rg16"<$>	**2f**	58	123	69
<$>\raster="rg18"<$>	**11**	34	64	64
<$>\raster="rg19"<$>	**12**	107	N.D.	N.D.
<$>\raster="rg20"<$>	**13**	65	N.D.	N.D.
<$>\raster="rg21"<$>	**14a**	69	N.D.	N.D.
<$>\raster="rg22"<$>	**14b**	55	N.D.	N.D.
<$>\raster="rg23"<$>	**17**	65	N.D.	N.D.
<$>\raster="rg24"<$>	**7**	64	N.D.	N.D.
<$>\raster="rg25"<$>	**8a**	71	87	81
<$>\raster="rg26"<$>	**8b**	82	N.D.	N.D.
<$>\raster="rg27"<$>	**3b** 2	92	N.D.	N.D.
<$>\raster="rg28"<$>	**3c** 2	74	N.D.	N.D.
<$>\raster="rg29"<$>	**4a** 2	62	N.D.	N.D.
<$>\raster="rg30"<$>	**4b** 2	91	N.D.	N.D.
<$>\raster="rg31"<$>	**4c** 2	51	6	3
<$>\raster="rg32"<$>	**5** 3	59	N.D.	N.D.

1 Prepared according to [28].

2 Prepared according to [29].

3 Prepared according to [30].

Residual *in vitro* activity of Pim-1 was determined in the presence of 10 µM concentrations of benzo[*cd*]azulenes dissolved in DMSO. Data were calculated as the percentage of Pim-1 autophosphorylation as compared with DMSO-treated controls. Residual cellular viabilities were determined by the MTT assay from FD/Neo and FD/Pim44 cells that had been cultured for 24 h without IL-3 in the presence of 0.1% DMSO or 5 µM inhibitors. Data were calculated as the percentage of viable cells in treated cultures as compared with DMSO-treated controls from at least two independent experiments with duplicate samples. N.D. means that viability was not determined.

To analyse the properties of the benzo[*cd*]azulene derivatives also in cell-based assays, FD/Neo and FD/Pim44 cells were cultured for 24 h in the presence of serum, but in the absence of IL-3. When cells were treated with 5 µM test compounds dissolved in DMSO, most of them did not have any effects on viability of either FD/Neo or FD/Pim44 cells or reduced it in both cell lines to a similar extent (Table **2**). Even though **2c** very efficiently inhibited the *in vitro* kinase activity of Pim-1, in cell-based assays it was far less potent with signs of some cytotoxicity. Indeed, only one of the newly synthesized compounds, **6a**, displayed similar properties as **1a** and **2f** and efficiently impaired the pro-survival advantage of Pim-1 overexpression in FD/Pim44 cells. However, **6a** also slightly affected the Neo-expressing control cells at the 5 µM concentration.

### Structure–activity Relationships of Novel Benzo[*cd*]azulenes

The structures and *in vitro* activities of compounds used for the structure-activity relationship are given in Figure **1**, Figure 2 and Tables **1** and **2**. In the heptafulvenic compound series with the isopropylidene substituent on the 7-membered ring, **1a** with the trifluoromethyl and methoxy substituents on 3- and 4-positions, respectively, displayed promising inhibition with residual Pim-1 kinase activity of 43%. Its regioisomer **1f** was nearly as active (residual activity 46%). The importance of the trifluoromethyl substituent was demonstrated by replacing it with a methyl group in **1g**, since this resulted in a complete loss of inhibitory activity (residual activity 105%). In cell-based assays, compound **1a** efficiently reduced Pim-1-dependent survival of FD/Pim44 cells (cell viability 65%) without marked effects on the FD/Neo control cells, and was therefore considered as an effective cell-permeable Pim-1-selective inhibitor. By contrast, the regioisomeric heptafulvene **1f** was completely ineffective in these assays and rather showed some signs of off-target cytotoxicity (FD/Neo 74%, FD/Pim44 97%).

The 4-phenol analog **1c** was found to be highly efficient *in vitro* with low residual Pim-1 activity (29%). However, since it showed chemical instability, it was excluded from cell-based assays. When the 4-hydroxy functionality of **1c** was replaced with a phenyl ring in **1e**, the residual Pim-1 activity was increased to 59% and no potency for this compound was observed within cells.

The tropones **2a** and **2f** were slightly less potent than the parental heptafulvenes **1a** and **1f**, with 74% and 58% residual *in vitro* activities of Pim-1, respectively. In cell-based assays **2a** was observed to pose some non-specific cytotoxicity affecting both FD/Neo and FD/Pim44 cells (80% and 81%, respectively). However, its regioisomer **2f** appeared to be as potent as **1a** and efficiently impaired the Pim-1-dependent survival of FD/Pim44 cells (69%) without any adverse effects on FD/Neo cells. When the trifluoromethyl substituent of **2a** was replaced with an ethoxycarbonyl group in **2d**, the kinase inhibitory activity was again completely lost (residual activity 98%), which was in line with the results on its heptafulvenic methyl analogue **1g**.

Tropone **2c** with a phenolic residue at 4-position was the most potent inhibitory compound *in vitro* with residual Pim-1 activity of only 11%, while its regioisomer **16** was not that efficient (residual activity 65%). Yet in cell-based assays **2c** was disappointingly far less potent than expected with some signs of off-target cytotoxicity (FD/Neo 85%, FD/Pim44 83%).

The five-member ring of **2a** was subjected to further modifications. This was possible through regioselective oxidation that yielded epoxide **13** with slightly stronger *in vitro* inhibition potential (residual activity 65%) than with the parental **2a** (residual activity 74%). The *cis*- and *trans*–diols **14b** and **14a** also had slightly better *in vitro* activities against Pim-1 (residual activities 55% and 69%, respectively). It should be mentioned here that both of these compounds showed signs of improved solubility into protic solvents (data not shown).

When the C = C double bond in the five-member ring of **2a** was reduced by catalytic hydrogenation, the non-planar alkane **12** was regioselectively obtained as a single compound. This compound did not have any effects against Pim-1 (residual activity 107%), suggesting that the planarity of the five-member ring is important for the observed inhibitory effects of the other compounds. While the quaternary aldehyde **11** displayed promising *in vitro* inhibition of Pim-1 (residual activity 34%), in cell-based assays it showed obvious signs of non-specific cytotoxicity (FD/Neo, FD/Pim44, both 64%).

The troponyl oxygen atoms on the 8-position of tropones **2a** and **2c** were also subjected to further modifications. The malononitrile groups in **6a** and **6b** restored the heptafulvenic structure and resulted in moderate to effective *in vitro* inhibition of Pim-1 (residual activities 60% and 41%, respectively). While the dicyanoheptafulvene **6a** reduced the survival of FD/Pim44 cells surprisingly well (viability 61%), it also turned out to be slightly cytotoxic for FD/Neo cells (78%), suggesting that such effective derivatives should be used at lower concentrations. By contrast, the phenolic derivative **6b** showed no efficacy in cell-based assays (FD/Pim44 93%), although *in vitro* it had been more potent than **6a**.

When the oximes **8a** and **8b** were transformed from the ketone carbonyl of **2a** and assayed separately as pure *Z*- and *E*-isomers, fairly mild inhibition of Pim-1 was observed *in vitro* (residual activities 71% and 82%, respectively). Similarly, the hydrazide derivative **7** showed only moderate inhibition (residual activity of 64%), as did also compound **5** representing the group of heterocyclic azulene derivatives (residual activity 59%).

In the series of benzo[*cd*]azulen-3-ones with a ketone carbonyl on the 3-position of the 6-membered ring, **3c** with a trifluoromethyl group was more effective *in vitro* against Pim-1 than **3b** with the methyl substituent (residual activities 74% and 92%, respectively). The subsequently dehydrated 4-methyl analog **4b** was also found to be inactive (residual activity 91%), but **4a** with no substituent at 4-position had moderate residual Pim-1 activity (residual activity 62%). The 4-trifluoromethyl analog **4c** displayed moderate *in vitro* activity against Pim-1, but was surprisingly effective when tested against Pim-3 (residual activities 51% and 24%, respectively). However, in the cell-based assays **4c** dramatically reduced the viability of both cell lines (FD/Neo 6%, FD/Pim44 3%), possibly due to its enhanced reactivity with various nucleophiles (data not shown) and/or its lack of target selectivity, as shown in Table 1.

## Discussion

Pim kinases have recently emerged as promising targets for therapy against both hematological malignancies and solid tumors. Therefore, there is increasing interest towards identification of potent and selective small molecule compounds inhibiting their activity. We have previously described synthesis of tricyclic benzo[*cd*]azulenes [28], [29], [30] and have now recognized that they possess kinase-inhibitory activity. Moreover, we have observed that under *in vitro* conditions, some of them show striking selectivity against Pim family kinases as compared with the 68 other protein kinases analysed. They are clearly more effective towards Pim-1 and Pim-3 than Pim-2, which correlates well with observations on several other compounds targeting the Pim family kinases [12], [27]. Since the amino acid sequences as well as structures of Pim family kinases are highly related to each other, their different sensitivities to inhibitory compounds remain to be explained.

The *in vitro* inhibitory activities of most benzo[*cd*]azulenes are not as impressive as with some other reported ATP-competitive compounds such as the pyrrolocarbazole **DHPCC-9**, which inhibits activities of Pim kinases already at low nanomolar concentrations [37]. Yet it is intriguing to notice that both types of compounds are equally efficient in cell-based assays, since both in our previous study [27] and in this study we have demonstrated them to abrogate the anti-apoptotic effects of Pim-1 in cytokine-deprived FDCP1 myeloid cells at low micromolar concentrations without any major signs of general cytotoxicity. Thus, it is possible that benzo[*cd*]azulenes are more permeable across cell membranes due to the planar but not ATP-mimetic lipophilic ring structure, less reactive with serum and other growth medium or intracellular constituents or otherwise reach their targets more efficiently to compensate for their lower *in vitro* activities. Since different types of Pim inhibitors have fairly distinct spectra of target specificities and since their inhibitory effects can be mimicked by using Pim-specific RNA interference reagents [27], it is highly likely that the observed effects of also benzo[*cd*]azulenes are due to their ability to selectively interfere with Pim activities.

Based on this study, the most potent benzo[*cd*]azulene structures to inhibit intracellular anti-apoptotic activities of Pim kinases were the heptafulvene 1a and the tropone **2f**, with low micromolar EC_50_ values. Further functional analyses indicated that **1a**-like benzo[*cd*]azulenes significantly reduce migration of adherent cancer cells derived from either prostate or squamocellular carcinomas. By contrast, such compounds do not significantly affect metabolic activity or viability of cancer cells or their levels of Pim protein expression. These results suggest that benzo[*cd*]azulenes or their derivatives have great potential in development of drugs against invasive tumors overexpressing Pim family members.

The ability of the benzo[*cd*]azulenes compounds such as **1a** to inhibit proliferation of EBV-transformed lymphoblastoid cell lines is also of interest, and suggests that these cells have become addicted to the EBV-induced expression of Pim kinases. Most people get infected with EBV before reaching adulthood, although usually the infection does not cause any major harm. However, since EBV remains latent in B-lymphocytes, it can reactivate itself later in immunocompromised individuals such as transplantation patients and cause aggressive lymphoproliferation and lymphoid tumours [38]. Therefore there is a clear demand to develop new, better tolerated drugs for the immunocompromised patients that are unusually sensitive to current chemotherapies, and need protection against EBV only transiently. Thus, the novel Pim-selective inhibitors or their derivatives may provide useful compounds for developing new drugs to restrict the effects of EBV in sensitized patients.

For this study, we have synthesized several novel benzo[*cd*]azulene structures and carried our structure-activity analyses to reveal the key features of both the known and novel compounds. These analyses have revealed that the CF_3_-substituent on the phenyl ring plays an essential role in effective inhibition of Pim-1 kinase. This was demonstrated with compounds bearing alternative substituents, such as those found in methyl and ethoxycarbonyl analogs **1g** and **2d**, both being inactive compounds. The presence of phenolic hydroxyl group on the six-membered ring of benzo[*cd*]azulenes was also important. Indeed, tropone **2c** and heptafulvenes **1c** and **6b**, bearing a phenol as a common structural feature at 4-position, showed efficient *in vitro* inhibitory activities against Pim-1. By contrast, the regioisomeric tropone **17** showed only modest efficiency as compared to the above-mentioned compounds.

Intriguingly, the *in vitro* activities of benzo[*cd*]azulenes did not always correlate with their efficacy in cell-based assays. While the tropone **2c** very efficiently inhibited Pim-1 activity *in vitro*, it was less potent in cells and also showed some signs of cytotoxicity. By contrast, **2f** displayed only mild effects *in vitro*, but was still almost as effective as **1a** in cell-based assays. The same fashion was seen with the dicyano-derived compound **6a**, which demonstrated good chemical stability over “normal” methyl-substituted heptafulvenes and which was the third most effective Pim-inhibitory compound in the cell-based assays. Thus, differences in solubility, stability and selectivity in addition to cell permeability may affect the biological outcomes, which are hard to predict just based on structure or even *in vitro* results. Ongoing optimization of additional benzo[*cd*]azulene derivatives is expected to further improve their efficacy as Pim-selective kinase inhibitors and anti-tumor drug candidates.

### Conclusions

In this study, we have identified and functionally characterized tricyclic benzo[*cd*]azulenes as new compounds capable of inhibiting protein kinase activity. Many of the described compounds are structurally novel, as also their synthesis routes, and several of them show selectivity towards Pim family kinases. While such benzo[*cd*]azulenes effectively inhibit *in vitro* autophosphorylation of Pim kinases, they are also able to enter the cells and efficiently impair intracellular anti-apoptotic and other activities of Pim kinases, as most strikingly demonstrated by the loss of Pim-dependent survival of cytokine-deprived myeloid cells. Furthermore, the Pim-inhibitory benzo[*cd*]azulenes significantly slow down migration of adherent cancer cells derived from either prostate or squamocellular carcinomas. In addition, they efficiently inhibit proliferation of lymphoblastoid cell lines (LCLs) that have been infected and immortalized by the Epstein-Barr virus. Taken together, benzo[*cd*]azulenes and their derivatives provide a new group of compounds that may be used not only as effective and selective research tools to investigate Pim functions, but also as promising scaffolds in development of small molecule therapies against Pim-overexpressing invasive tumors and other tumorigenic disorders.

## Materials and Methods

### Kinase Selectivity Assays

The selectivity of the compounds **1a**, **1e**, **2a**, **2f**, **4b** and **4c** was tested against 71 kinases on a commercial basis in a kinase platform at the Division of Signal Transduction Therapy, University of Dundee, UK. Assays were run at ATP concentrations, which were close to the *K*_m_ value of each kinase. One concentration (10 µM) of the compounds was used with each kinase with duplicate wells [30]. The data is expressed as the percentage of residual kinase activity. Due to the two-point inhibition data, the uncertainty values were large and this is why only residual activity <50% was considered significant.

### *In vitro* Phosphorylation Assays

Wild-type human Pim-1 protein produced in bacteria as a GST-fusion protein was purifed with glutathione sepharose beads (GE Healthcare) and cleaved with PreScission protease (GE Healthcare) according to manufactureŕs instructions. For each kinase reaction, 0.5–1 µg of Pim-1 protein was preincubated for 10 min with DMSO-dissolved compounds at a final inhibitor concentration of 10 µM. DMSO alone was used in control reactions. Radioactive kinase reactions were performed in a buffer containing 15 mM Pipes (pH 7.4), 5.5 mM MnCl_2_, and 15 µM ATP with a specific activity of 150 µCi/mL for 15 min at 30°C. Reactions were stopped by boiling in Laemmli sample buffer for 5 min at 95°C. Phosphorylated proteins were resolved in 10% SDS-PAGE and stained with Coomassie blue (PAGE-BLUE, Fermentas) to confirm equal loading. Radioactivity of the samples was analysed by autoradiography and quantitated by the MCID M5+ Image Analyzer (InterFocus, UK). Data were calculated as the percentage of residual Pim-1 kinase activity as compared with DMSO-treated controls.

### Cell Lines and Culture Conditions

The murine IL-3-dependent myeloid FDCP1 cell lines [13], the lymphoblastoid cell lines (LCLs) infected and immortalized by Epstein-Barr virus (EBV) [20] and the head and neck squamocellular carcinoma cell line UT-SCC-12A [15] have all been described previously. The human androgen-independent prostate epithelial adenocarcinoma cell line PC-3 was obtained from the American Type Culture Collection. FDCP-1, LCL and PC-3 cells were grown in RPMI-1640 medium and UT-SCC-12A cells were grown in DMEM medium. All media were supplemented with 10% fetal bovine serum, 2 mM L-glutamine, 100 U/mL penicillin and 100 µg/mL streptomycin. In addition, 1% non-essential amino acids were added to UT-SCC-12A cell cultures and 10% WEHI-conditioned medium was used as the source of IL-3 for FDCP1 cell lines.

### Cell Viability Assays

For viability assays, FDCP1 cell lines were seeded onto 96-well plates at 2×10^5^ cells/mL and grown with or without IL-3 for different time-points. Varying concentrations of the test compounds dissolved in DMSO were added to the cellular culture medium, while 0.1% DMSO was added to control cell samples. Cells were incubated for 24 h, after which their viabilities were analyzed either by MTT assays or Trypan blue staining, as described previously [27].

Lymphoblastoid cell lines (LCLs) cells were seeded onto 12-well plates at either 1 or 3×10^5^ live cells/mL. Cells were then treated with 10 µM test compounds or 0.1% DMSO. For each treatment, there were two parallel samples. Cell density was kept under 10^6^/mL over the whole experiment, and additional medium and DMSO or test compounds were added when density was getting higher. The amounts of live cells excluding Trypan blue were counted on alternate days.

### Western Blotting

Expression levels of 50 µg aliquots of proteins were measured from cell pellets by Western blotting as described previously [27] using the following antibodies: anti-Pim-1 (1∶10000 dilution of EP2645Y; Abcam), anti-Pim-2 (1∶1000 of D1D2; Cell Signaling Technology), anti-Pim-3 (1∶1000 of D17C9; Cell Signaling Technology) and anti-GAPDH (1∶20000; Sigma-Aldrich) antibodies.

### Wound Healing Assays

Cells were plated on 24-well plates, allowed to attach for 24 h, and then treated with either 0.1% DMSO or 10 µM DMSO-dissolved test compounds. Two hours later, scratch wounds were made with a sterile 200 µL pipette tip. Photographs were taken using the Zeiss Stereo Lumar-V12 microscope with the AxioVision Rel.4.8 software with 35-fold enlargement. Wounds were outlined and the wound areas were measured by the ImageJ software (Wayne Rasband, NIH, USA).

### Statistical Analyses

Microsoft Excel was used to calculate wound healing percentages and statistical significance of data (t-test: paired two samples for means). Results were interpreted as highly significant*** (p<0.001), significant** (p<0.01), weakly significant* (p<0.05) or not significant**^ns^** (p>0.05). IC_50_ values of test compounds in FDCP-1 cells were determined using nonlinear regression fitting with the GraphPad Prism v.5.0. Error bars in all graphs represent SD values.

## Supporting Information

Document S1 **Supporting Information.**
(DOC)

Movie S1 **PC-3 cell migration after DMSO treatment).**
(AVI)

Movie S2 **PC-3 cell migration after Pim inhibition by the benzo[cd]azulene 1a.**
(AVI)
