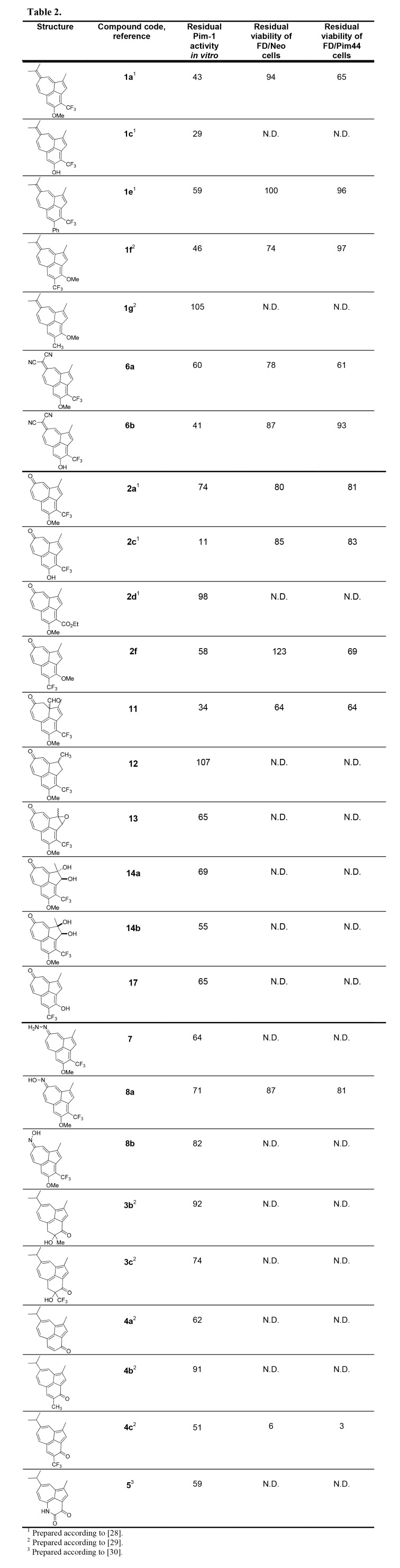# Correction: Tricyclic Benzo[*cd*]azulenes Selectively Inhibit Activities of Pim Kinases and Restrict Growth of Epstein-Barr Virus-Transformed Cells

**DOI:** 10.1371/annotation/f0b00bc7-fab8-4186-967a-5fd49a857013

**Published:** 2013-10-17

**Authors:** Alexandros Kiriazis, Riitta L. Vahakoski, Niina M. Santio, Ralica Arnaudova, Sini K. Eerola, Eeva-Marja Rainio, Ingo B. Aumüller, Jari Yli-Kauhaluoma, Päivi J. Koskinen

Compound 11 was erroneously omitted from Table 2. Please see the correct Table 2 here: 

**Figure:**