# Supplementary material for: Tricyclic Benzo[cd]azulenes Selectively Inhibit Activities of Pim Kinases and Restrict Growth of Epstein-Barr Virus-Transformed Cells
Source: PLoS One. 2013 Feb 6;8(2):e55409. doi: 10.1371/journal.pone.0055409 (PMC3566155; doi:10.1371/journal.pone.0055409)
Supplement: Document S1 — Supporting Information. (DOC) [file pone.0055409.s001.doc]

**Supporting Information**

**Tricyclic Benzo[*cd*]azulenes Selectively Inhibit Activities of Pim Kinases and Restrict Growth of Epstein-Barr Virus-transformed Cells**

##### **Alexandros Kiriazis, Riitta L. Vahakoski, Niina M. Santio, Ralica Arnaudova, Sini K. Eerola, Eeva-Marja Rainio, Ingo B. Aumüller, Jari Yli-Kauhaluoma, Päivi J. Koskinen**

**Additional Movie files (Movie S1 and Movie S2) – Movie S1 represents control experiment and Movie S2 the effects of 1a on prostate cancer cell migration.** PC-3 cells were plated on 24-well Essen ImageLock plates and wounded 24 h later by the Essen WoundMaker^TM^ with sterile 10 µL pipette tips. Thereafter cells were treated with either 0.1% DMSO or 10 µM **1a**. The scratch wound movie images were acquired at 2 hour intervals by the Essen IncuCyte^TM^ Scratch Wound software. After acquisition, images were combined and movie files were created by ImageJ. Shown are representative wound healing movies of PC-3 cells treated with DMSO (Movie S1) or the Pim-selective inhibitor **1a** (Movie S2).

**Chemistry experimental details**

**General**

All reactions were carried out using commercially available starting materials (Aldrich, Schnelldorf, Germany and Fluka, Buchs, Switzerland) and solvents without further purification under an argon atmosphere. Tetrahydrofuran (THF) and diethyl ether (Et_2_O) were distilled under argon with sodium/benzophenone ketyl and dichloromethane with calcium hydride. Column chromatography was performed with Merck 230−400 mesh silica gel. The melting points were recorded with an Electrothermal capillary tube melting point apparatus (Electrothermal Engineering, Essex, UK) and are uncorrected. ^1^H NMR (300 MHz) and ^13^C NMR (75 MHz) spectra in CDCl_3_, DMSO-*d*_6_, acetone-*d*_6_ or CD_3_OD at ambient temperature were recorded on a Varian Mercury *Plus* 300 spectrometer (Varian, Palo Alto, CA, USA). The chemical shifts were reported in parts per million (ppm) and on the *δ* scale from tetramethylsilane as an internal standard. The coupling constants *J* are quoted in Hertz (Hz). GC–MS analyses were performed on a Hewlett-Packard 5890 series gas chromatograph (Hewlett-Packard Company, Palo Alto, CA, USA) equipped with an HP-5970 mass selective detector. LC-MS analyses were performed by the use of an HP1100 instrument (Hewlett-Packard Company, Palo Alto, CA, USA) with UV detector (λ 210 nm) and Esquire LC spectrometer (Bruker Daltonik, Bremen, Germany) with ESI ion source. Signal separation was carried out by use of a Waters XBridge C18 2.1 mm × 10 mm, 2.5 μm pre-column with Waters and XBridge C18 2.5 μm, 2.1 mm × 50 mm column. High resolution mass spectra (HRMS) were measured on a Waters Synapt G2 (Waters Corporation, Massachusetts, USA). Elemental analyses (CHN) were obtained from Robertson Microlit Laboratories, Inc., Madison, New Jersey, USA.

**2-[4-Methoxy-1-methyl-3-(trifluoromethyl)-8*H*-benzo[*cd*]azulen-8-ylidene] malo-nonitrile 6a.**

To a solution of tropone **2a** [28] (0.10 g, 0.34 mmol) in methanol (4 mL) was added malononitrile (68 mg, 1.02 mmol) and ammonium acetate (31 mg, 0.40 mmol) and the reaction mixture was refluxed for 3 d under argon atmosphere. The solvents were evaporated by a rotary evaporator, and the obtained crude product was purified by column chromatography on silica gel (eluent: PhMe) to yield **6a** (52 mg, 45%) as red needles. M. p. 227–228 °C; ^1^H NMR (300 MHz, CDCl_3_): *δ* 7.42 (1H, d, *J* = 2.6 Hz), 7.28 (1H, dd, *J* = 2.6 Hz, 12.0 Hz), 7.10 (1H, d, *J* = 12.0 Hz), 6.67 (1H, s), 3.98 (3H, s), 2.26 (3H, s); ^13^C NMR (75 MHz, CDCl_3_): *δ* 160.4, 159.9, 150.7, 143.8, 143.4, 140.1, 135.7, 132.2 (q, *J* = 4.6 Hz), 129.5, 127.6, 124.9, 123.5 (q, *J* = 272.5 Hz), 115.9 (q, *J* = 31.9 Hz), 114.1, 114.1, 110.3, 78.8, 56.8, 12.8 ppm; Anal. calc. for C_19_H_11_F_3_N_2_O (340.08): C, 67.06; H, 3.26; N, 8.23. Found: C, 67.13; H, 3.28; N, 8.12. LC–MS: [M+H]^+^ *m*/*z* 341.

**2-[4-Hydroxy-1-methyl-3-(trifluoromethyl)-8*H*-benzo[*cd*]azulen-8ylidene]malo- nonitrile 6b.**

To a solution of tropone **2c** [28] (0.10 g, 0.36 mmol) in methanol (10 mL) was added malononitrile (0.060 g, 0.90 mmol, 2.5 eq) and ammonium acetate (69 mg, 0.90 mmol) and the reaction mixture was heated at 70 °C for 4 d under argon atmosphere. The reaction mixture was cooled down and ethyl acetate (10 mL) and water (10 mL) were added. The phases were separated and the aqueous phase was extracted with EtOAc (2 × 10 mL). The combined organic phases were dried over anhydrous Na_2_SO_4_, filtered and evaporated to dryness. The obtained crude product was purified by column chromatography on silica gel (eluent: EtOAc/*n*-hexane 1:1) to yield **6b** (26 mg, 22%) as brown-orange solid. M. p. 252 °C (dec.); ^1^H NMR (300 MHz, acetone-*d*_6_): *δ* 7.43 (1H, d, *J* = 2.4 Hz), 7.39 (1H, d, *J* = 12.0 Hz), 7.25 (1H, dd, *J* = 2.4 Hz, 12.0 Hz), 7.10 (1H, d, *J* = 1.5 Hz), 7.02 (1H, s), 2.31 (3H, d, *J* = 1.5 Hz); ^13^C NMR (75 MHz, acetone-*d*_6_): *δ* 160.6, 159.5, 151.0, 144.4, 144.1, 141.2, 136.9, 132.2 (q, *J* = 4.3 Hz), 129.6, 126.7, 125.3, 124.9 (q, *J* = 270.5 Hz), 116.3, 114.8 (q, *J* = 31.9 Hz), 114.7, 114.7, 79.2, 12.4 ppm; Anal. calc. for C_18_H_9_F_3_N_2_O (326.07): C, 66.26; H, 2.78; N, 8.59. Found: C, 66.25; H, 2.97; N, 8.29; LC–MS: [M+H]^+^ *m*/*z* 327.

**(*E*, *Z*)-4-Methoxy-1-methyl-3-(trifluoromethyl)-8*H*-benzo[*cd*]azulen-8-ylidene hydrazide 7.**

Tropone **2a** (0.300 g, 1.03 mmol) was dissolved in anhydrous methanol (8 mL) and hydrazine monohydrate (102 µL, 2.06 mmol, 2.0 eq) was added. The reaction mixture was refluxed for 20 h under argon atmosphere. TLC analysis from the reaction mixture indicated a formation of two red-orange products. The reaction mixture was cooled to room temperature and dichloromethane (20 mL) and water (10 mL) were added. The phases were separated and the aqueous phase was extracted with CH_2_Cl_2_ (10 mL). The combined organic phases were dried over anhydrous Na_2_SO_4_, filtered and evaporated to dryness. The obtained crude product was purified by column chromatography on silica gel (eluent: EtOAc/*n–*hexane 3:1) to yield hydrazide **7** as an orange powder (137 mg, 44%). The ^1^H NMR indicated two diastereomers (*Z/E*) of the desired hydrazide product with an approximate ratio of 1:1. ^1^H NMR (300 MHz, DMSO-*d*_6_): *δ* 7.82 (2H, s), 7.72 (2H, s), 6,86 (1H, d, *J* = 2.1 Hz), 6.72–6.67 (2H, m), 6.67–6.60 (2H, m), 6.53 (1H, s), 6.49 (1H, d, *J* = 2.1 Hz), 6.31 (1H, d, *J* = 12.9 Hz), 6.21 (1H, d, *J* = 2.1, 12.9 Hz), 5.99 (1H, d, *J* = 12.9 Hz), 3.83 (3H, s), 3.82 (3H, s), 2.17 (3H, s), 2.11 (3H, s) ppm; Anal. calc. for C_16_H_13_F_3_N_2_O (307.08): C, 62.74; H, 4.28; N, 9.15. Found: C, 63.03; H, 4.10; N, 8.67. LC–MS: [M+H]^+^ *m*/*z* 307.

**(*E*)-4-Methoxy-1-methyl-3-(trifluoromethyl)-8*H*-benzo[*cd*]azulen-8-one oxime 8a** and **(*Z*)-4-methoxy-1-methyl-3-(trifluoromethyl)-8*H*-benzo[*cd*]azulen-8-one oxime 8b.**

Tropone **2a** (0.300 g, 1.03 mmol) was dissolved in isopropanol–water mixture (3:1, 20 mL) and hydroxylamine hydrochloride (427 mg, 6.15 mmol, 6 eq) and ammonium acetate (474 mg, 6.15 mmol, 6 eq) were added. The reaction mixture was heated for 5 d at 80 °C under argon atmosphere. TLC analysis from the reaction mixture indicated a formation of two closely moving products and unreacted starting material. The reaction mixture was cooled down and dichloromethane (50 mL) and water (15 mL) were added. The phases were separated and the aqueous phase was extracted with dichloromethane (25 mL). The combined organic phases were dried over anhydrous Na_2_SO_4_, filtered and evaporated to dryness. The obtained crude product was purified by column chromatography on silica gel (eluent: EtOAc/cyclohexane 1:2) to yield less polar fraction as a yellow solid. The oxime product was further purified by recrystallization from EtOAc/*n*-hexane to give **8a** (133 mg, 42%) as bright-yellow needles. M. p. 240 °C (dec.); ^1^H NMR (300 MHz, DMSO-*d*_6_): *δ* 12.13 (1H, br s), 7.04 (1H, dd, *J* = 2.1 Hz, 12.9 Hz), 6.87 (1H, s), 6.79 (2H, m), 6.67 (1H, d, *J* = 12.9 Hz), 3.88 (3H, s), 2.17 (3H, s); ^13^C NMR (75 MHz, DMSO-*d*_6_): *δ* 157.9, 152.5, 143.0, 141.1, 139.9, 135.2, 132.7, 130.0, 126.9, 126.6 (q, *J* = 4.5 Hz), 124.4 (q, *J* = 272.0 Hz), 121.4, 110.3, 109.5 (q, *J* = 30.0 Hz), 56.5, 12.3 ppm; Anal. calc. for C_16_H_12_F_3_NO_2_ (307.08): C, 62.54; H, 3.94; N, 4.56. Found: C, 62.45; H, 3.82; N, 4.53; LC–MS: [M+1]^+^ *m*/*z* 308.

The more polar fraction was isolated as an orange solid and was further purified by recrystallization from EtOAc/*n*-hexane to yield **8b** (93 mg, 29%) as orange needles. M. p. 238 °C (dec.); ^1^H NMR (300 MHz, DMSO-*d*_6_): *δ* 12.04 (1H, br s), 7.30 (1H, d, *J* = 1.8 Hz, H-9), 6.84 (1H, s), 6.81 (1H, s), 6.53 (1H, d, *J* = 12.9 Hz, H-6), 6.46 (1H, dd, *J* = 1.8 Hz, 12.9 Hz, H-7) 3.87 (3H, s), 2.15 (3H, s); ^13^C NMR (75 MHz, DMSO-*d*_6_): *δ* 158.5, 152.6, 143.7, 142.2, 141.7, 141.6, 136.7, 132.5, 128.6 (q, *J* = 4.5 Hz), 125.7, 124.3 (q, *J* = 272.0 Hz), 117.5, 109.7, 109.2 (q, *J* = 30.0 Hz), 56.5, 12.3 ppm; Anal. calc. for C_16_H_12_F_3_NO_2_ (307.08): C, 62.54; H, 3.94; N, 4.56. Found: C, 62.58; H, 3.77; N, 4.59. NOESY correlation between oxime hydroxy group (12.04 ppm) and aromatic proton H-9 (7.30 ppm) was found. The stereochemistry of the oxime **8b** was assigned to be (*Z*). LC–MS: [M+H]^+^ *m*/*z* 308.

**(±)-4-Methoxy-1-methyl-8-oxo-3-(trifluoromethyl)-9,9*a*-dihydro-8*H*-benzo[*cd*] azulene-9a-carbaldehyde 11.**

To a solution of (methoxymethyl)triphenylphosphonium chloride (127 mg, 0.37 mmol) in anhydrous THF (6 mL) was added *n*-butyllithium solution (2.5 M in hexanes, 0.16 mL, 0.39 mmol) at –78 °C under argon atmosphere. After keeping the formed ylide at 0 °C over 30–40 min it was recooled to –78 °C and then a solution of tropone **2a** (0.10 g, 0.34 mmol) in anhydrous THF (2 mL) was added dropwise to the reaction mixture under argon atmosphere and let to warm up to room temperature overnight. The reaction mixture was quenched with aqueous HCl (2 M, 5 mL) and diethyl ether (15 mL) was added. The aqueous phase was extracted with diethyl ether (2 × 10 mL) and the combined organic phases were dried over anhydrous Na_2_SO_4_, filtered and evaporated to dryness to give the crude product as a bright orange oil. It was purified by column chromatography on silica gel (eluent: EtOAc/*n*-hexane from 1:2 to 1:1) to give **11** (42 mg, 38%) as a yellow solid. M. p. 149 °C (dec); ^1^H NMR (300 MHz, CDCl_3_): *δ* 8.23 (1H, d, *J* = 1.5 Hz), 7.18 (1H, m), 6.93 (1H, d, *J* = 12.6 Hz), 6.79 (1H, s), 6.27 (1H, dd, *J* = 1.5, 12.6 Hz), 3.96 (3H, s), 3.42 (1H, dd, *J* = 1.5 Hz, 16.8 Hz), 2.49 (1H, dd, *J* = 1.5 Hz, 16.8 Hz), 1.99 (3H, d, *J* = 1.5 Hz) ppm; ^13^C NMR (75 MHz, CDCl_3_): *δ* 196.1, 193.8, 158.6 (q, *J* = 2.3), 148.7, 143.8 (q, *J* = 1.7 Hz), 137.3, 134.6, 134.4, 132.7, 130.5 (q, *J* = 4.6 Hz), 124.0 (q, *J* = 272.0 Hz), 111.3, 110.1 (q, *J* = 31.0 Hz), 65.3, 56.8, 40.9, 13.1 ppm; NOESY spectra showed correlations between formyl hydrogen (8.23 ppm) and methyl group C-1 (1.99 ppm); Anal. calc. for C_17_H_13_F_3_O_3_ (322.08): C, 63.36; H, 4.07. Found: C, 63.12; H, 4.01; LC–MS: [M+H]^+^ *m*/*z* 323.

**(±)-4-Methoxy-1-methyl-3-(trifluoromethyl)-1*H*-benzo[*cd*]azulen-8(2*H*)-one 12.**

Tropone **2a** (0.20 g, 0.69 mmol) was dissolved in ethyl acetate (10 mL) and palladium 10 w. % in charcoal (cat.) was added. The reaction mixture was cooled to 0 °C and then allowed to react with hydrogen (H_2_ balloon) for 50 min. TLC analysis from the reaction mixture showed disappearance of the yellow starting material and a formation of slower moving colorless main product together with side products. The reaction mixture was filtered off through a pad of Celite and the filtrate was then evaporated by a rotary evaporator. The crude product was purified by column chromatography on silica gel (eluent: EtOAc/*n*–hexane 3:2) to yield **12** (80 mg, 40%) as off-white crystalline solid. M. p. 150–152 °C; ^1^H NMR (300 MHz, DMSO-*d*_6_): *δ* 7.59 (1H, d, *J* = 12.3 Hz), 7.61 (1H, s), 6.77 (1H, dd, *J* = 2.3, 12.3 Hz), 6.60 (1H, m), 4.01 (3H, s), 3.67–3.54 (1H, m), 3.44–3.33 (1H, m), 2.93 (1H, td, *J* = 3.0 Hz, 18.0 Hz), 1.30 (3H, d, *J* = 7.2 Hz) ppm; ^13^C NMR (75 MHz, DMSO-*d*_6_): *δ* 185.6, 162.2, 159.5 (q, *J* = 2.0 Hz), 149.5 (q, *J* = 2.0 Hz), 138.1, 138.0, 137.9, 132.2, 127.0, 124.0 (q, *J* = 273.0 Hz), 115.3 (q, *J* = 30.0 Hz), 114.0, 57.0, 38.8, 38.4 (q, *J* = 4.0 Hz), 21.4 ppm; Anal. calc. for C_16_H_13_F_3_O_2_ (294.09): C, 65.30; H, 4.45. Found: C, 65.15; H, 4.31; LC–MS: [M+H]^+^ *m*/*z* 295.

**(±)-2-Methoxy-7*b*-methyl-1-(trifluoromethyl)-7*b*,8*a*-dihydro-6*H*-benzo[3,4] azuleno[1,2-*b*]oxiren-6-one 13.**

To a solution of tropone **2a** (0.300 g, 1.03 mmol) in dichloromethane (16 mL) was added *m*-chloroperoxybenzoic acid (max 77%, 686 mg, 3.06 mmol, 3.0 eq) at 0 °C and the reaction mixture was left to stir for 5 d at room temperature. After 5 d TLC analysis from the reaction mixture showed a formation of slower moving colorless main product and unreacted starting material. 10% aqueous solution of sodium sulphite (5 mL) was added followed by addition of aqueous NaOH (1 M, 10 mL) and dichloromethane (30 mL). The phases were separated and the organic phase was washed with NaOH (1 M, 10 mL) and water (20 mL). The combined organic phases were dried over anhydrous Na_2_SO_4_, filtered and evaporated to dryness. The obtained crude product was purified by column chromatography on silica gel (eluent: EtOAc/*n*-hexane 1:1) to yield **13** (132 mg, 42%) as an off-white solid. M. p. 203–205 °C (dec.); ^1^H NMR (300 MHz, CDCl_3_): *δ* 7.28 (H, d, *J* = 12.6 Hz), 7.06 (1H, s), 6.95 (1H, d, *J* = 2.7 Hz), 6.87 (1H, dd, *J* = 2.7 Hz, 12.6 Hz), 4.81 (1H, q, *J* = 2.7 Hz), 4.01 (3H, s), 1.87 (3H, s) ppm; ^13^C NMR (75 MHz, CDCl_3_): *δ* 186.9, 159.1 (q, *J* = 1.7 Hz), 150.2, 143.7 (q, *J* = 2.9 Hz), 138.7, 138.5, 138.3, 132.5, 130.6, 123.6 (q, *J* = 273.0 Hz), 119.6 (q, *J* = 31.9 Hz), 114.0, 65.4, 62.3 (q, *J* = 5.1 Hz), 56.9, 14.4 ppm; Anal. calc. for C_16_H_11_F_3_O_3_ (308.07): C, 62.34; H, 3.60. Found: C, 62.25; H, 3.35; LC–MS: [M+H]^+^ *m*/*z* 309.

**(±)-1,2-*trans*-Dihydroxy-4-methoxy-1-methyl-3-(trifluoromethyl)-1*H*-benzo[*cd*] azulen-8(*2H*)-one *rac*-14a.**

Epoxide **13** (0.060 g, 0.19 mmol) was dissolved in a mixture of water and 1,4-dioxane (1:4, 2–3 mL) and perchloric acid (70% in water, 15–20 drops) was added at room temperature. The reaction mixture was stirred for 4 d and TLC analysis from the reaction mixture showed a formation of slower moving colorless product and complete consumption of starting material. A solution of NaOH (1 M, 5 mL) was added for neutralization followed by addition of EtOAc (10 mL). The aqueous phase was extracted with EtOAc (2 × 10 mL) and the combined organic phases were dried over anhydrous Na_2_SO_4_, filtered and evaporated to dryness. The obtained crude product was purified by column chromatography on silica gel (eluent: EtOAc/*n*-hexane 6:1) to yield *rac*-**14a** (55 mg, 89%) as a white solid which was further purified by recrystallization from isopropanol–*n*-hexane to give white needles. M. p. 224–226 °C (dec.); ^1^H NMR (300 MHz, DMSO-*d*_6_): *δ* 7.73 (1H, s), 7.67 (1H, d, *J* = 12.9 Hz), 6.81 (1H, dd, *J* = 2.4, 12.9 Hz), 6.67 (1H, d, *J* = 2.4 Hz), 5.92 (1H, d, *J* = 7.8 Hz), 5.59 (1H, s), 4.96 (1H, m), 4.03 (3H, s), 1.46 (3H, s) ppm; ^13^C NMR (75 MHz, DMSO-*d*_6_): *δ* 186.8, 159.6, 158.7, 148.8, 138.4, 138.2, 137.7, 130.8, 127.4, 123.6 (q, *J* = 273.0 Hz), 116.6 (q, *J* = 31.0 Hz), 115.6, 80.8, 79.4, 57.1, 21.6 ppm; Anal. calc. for C_16_H_13_F_3_O_4_ (326.08): C, 58.90; H, 4.02. Found: C, 58.80; H, 3.78; LC–MS: [M+H]^+^ *m*/*z* 327.

**(±)-1,2-*cis*-Dihydroxy-4-methoxy-1-methyl-3-(trifluoromethyl)-1*H*-benzo[*cd*] azulen-8(*2H*)-one *rac*-14b.**

Tropone **2a** (0.20 g, 0.69 mmol) was dissolved in a mixture of water and THF (1:3, 9 mL) and *N*–methylmorpholine *N*–oxide (121 mg, 1.04 mmol, 1.5 eq) and osmium tetroxide (4% in water, 490 μL, 0.08 mmol, 0.12 eq) were added at room temperature and the reaction mixture was stirred for 3 d. TLC analysis from the reaction mixture showed a disappearance of staring material and a formation of slower moving colorless product. 10% aqueous solution of sodium sulphite (20 mL) and EtOAc (20 mL) were added. The aqueous phase was extracted with EtOAc (2 × 10 mL) and the combined organic phases were dried over anhydrous Na_2_SO_4_, filtered and evaporated to dryness. The obtained crude product was purified by column chromatography on silica gel (eluent: EtOAc/*n*-hexane 4:1) to yield *rac*-**14b** (115 mg, 51%) as a white solid. M. p. 172–174 °C; ^1^H NMR (300 MHz, DMSO-*d*_6_): *δ* 7.73 (1H, s), 7.67 (1H, d, *J* = 12.9 Hz), 6.81 (1H, dd, *J* = 2.4, 12.9 Hz), 6.67 (1H, d, *J* = 2.4 Hz), 5.92 (1H, d, *J* = 7.8 Hz), 5.59 (1H, s), 4.96 (1H, m), 4.03 (3H, s), 1.46 (3H, s) ppm; ^13^C NMR (75 MHz, DMSO-*d*_6_): *δ* 186.2, 160.6, 159.6 (q, *J* = 2.0 Hz), 146.6 (q, *J* = 1.7 Hz), 138.3, 138.0, 138.0, 129.6, 127.6, 123.5 (q, *J* = 273.0 Hz), 116.8 (q, *J* = 31.0 Hz), 115.8, 78.6, 76.2, 57.1, 28.8 ppm; Anal. calc. for C_16_H_13_F_3_O_4_ (326.08): C, 58.90; H, 4.02. Found: C, 59.15; H, 3.89; LC–MS: [M+H]^+^ *m*/*z* 327.

**3-Methoxy-1-methyl-4-(trifluoromethyl)-8*H*-benzo[*cd*]azulen-8-one 2f.**

Heptafulvene **1f** [29] (224 mg, 0.70 mmol) was dissolved in dichloromethane (15 mL) and cooled down to 0 °C under argon atmosphere. *m-*Chloroperoxybenzoic acid (max. 77%, 394 mg, 1.76 mmol, 2.5 eq) was added and the mixture was stirred at 0 °C until TLC analysis indicated the complete consumption of the starting material (1–2 h). During this period the color of the reaction mixture changed from red-orange to orange-yellow. 10% aqueous solution of sodium sulphite (10 mL) was added followed by addition of aqueous NaOH (1 M, 10 mL) and dichloromethane (30 mL). The phases were separated and the organic phase was washed with NaOH (1 M, 10 mL) and water (20 mL). The combined organic phases were dried over anhydrous Na_2_SO_4_, filtered and evaporated to dryness. The crude product was purified by column chromatography on silica gel (eluent: EtOAc/*n-*hexane 6:7). The product **2f** was obtained as thin yellow needles (174 mg, 85%). M. p. 186 °C; ^1^H NMR (300 MHz, DMSO–*d_6_*): *δ* 8.06 (1H, s), 7.72 (1H, d, *J* = 12.3 Hz), 7.47 (1H, d, *J* = 1.5 Hz), 7.19 (1H, d, *J* = 1.8 Hz), 6.83 (1H, dd, *J* = 1.8 Hz, 12.3 Hz), 4.19 (3H, s), 2.27 (3H, d, *J* = 1.5 Hz) ppm; ^13^C NMR (75 MHz, DMSO–*d_6_*): *δ* 186.3, 151.7, 147.2, 139.2, 138.2, 137.6, 134.6, 134.1, 130.9 (q, *J* = 5 Hz), 130.3, 129.5, 126.1, 123.1 (q, *J* = 271 Hz), 122.9 (q, *J* = 30 Hz), 62.0, 12.2 ppm; Anal. calc. for C_16_H_11_F_3_O_2_ (292.07): C, 65.76; H, 3.79. Found: C, 65.83; H, 3.74; LC–MS: [M+H]^+^ *m/z* 293.

***tert*-Butyldimethyl[(1-methyl-8-(propan-2-ylidene)-4-(trifluoromethyl)-8*H*-benzo [*cd*]azulen-3-yl)oxy]silane 16.**

Benzo[*cd*]azulen-3-one **4c** [29] (150 mg, 0.49 mmol) was dissolved under argon atmosphere in anhydrous tetrahydrofuran (8 mL) and hydrochloric acid (conc.) (15 μL) is added. After the color changed from green to dark red (20–40 min), sodium hydride (60% suspension in mineral oil, 59 mg, 2.49 mmol, 5.0 eq) and *tert*-butyldimethylsilyl chloride (186 mg, 1.25 mmol, 3.0 eq) are added. Stirring is continued until TLC analysis indicates complete consumption of the starting material and a formation of the orange product. Ethyl acetate (15 mL) was added and the resulting solution was washed twice with saturated sodium hydrogen carbonate solution (10 mL) and twice with water (10 mL). The combined organic phases were dried over anhydrous Na_2_SO_4_, filtered and evaporated to dryness. The crude enol ether **16** was purified by column chromatography on silica gel (eluent: cyclohexane). The product **16** was obtained as bright-red solid (172 mg, 84%). M. p. 108–110 °C; ^1^H NMR (300 MHz, CDCl_3_): *δ* 6.94 (1H, d, *J* = 1.8 Hz), 6.82 (1H, s), 6.51 (1H, s), 6.14 (1H, dd, *J =* 1.8 Hz, 12.9 Hz), 5.90 (1H, d, *J =* 12.9 Hz), 2.11 (3H, d, *J* = 1.2 Hz), 1.95 (3H, s), 1.88 (3H, s), 1.01 (9H, s), 0.22 (6H, s); ^13^C NMR (75 MHz, CDCl_3_): *δ* 145.2, 140.2, 138.7, 138.5, 137.7, 133.7, 133.5, 131.0, 128.1, 128.0, 127.1, 124.8 (q, *J* = 5 Hz), 124.7, 124.1 (q, J = 271 Hz), 121.0 (q, *J* = 29 Hz), 29.9, 26.1, 26.1, 26.1, 22.3, 22.0, 12.8, -3.15, -3.16 ppm; Anal. calc. for C_24_H_29_F_3_OSi (418.19): C, 68.87; H, 6.98. Found: C, 70.52; H, 7.78; GC–MS: [M]^+^ *m/z* 418; HRMS-ESI *m/z*: calc. for [M + Na]^+^: 441.1837, found 441.2029.

**3-Hydroxy-1-methyl-4-(trifluoromethyl)-8*H*-benzo[*cd*]azulen-8-one 17.** The enol ether **16** (174 mg, 0.42 mmol) was dissolved in dichloromethane (10 mL) and cooled down to 0 °C under argon atmosphere. *m-*Chloroperoxybenzoic acid (max. 77%, 208 mg, 0.92 mmol, 2.2 eq) and sodium hydrogen carbonate (1.20 mmol, 106 mg, 3.0 eq) were added and the mixture was stirred at 0 °C for 1 h. 10% aqueous solution of sodium sulphite (10 mL) was added followed by addition of sat. solution of aqueous sodium hydrogen carbonate (10 mL) and dichloromethane (15 mL). The phases were separated and the organic phase was washed with sat. solution of aqueous sodium hydrogen carbonate (10 mL) and water (15 mL). The combined organic phases were dried over anhydrous Na_2_SO_4_, filtered and evaporated to dryness to give the crude oxidation product which was used for the next step without further purification. The crude silyl enol ether was dissolved in anhydrous THF (4 mL) followed by addition of *n*-tetrabutylammonium fluoride solution (1.0 M in THF) (0.52 mL, 0.52 mmol) under argon atmosphere. After 2 h TLC showed disappearance of starting material. Ethyl acetate (10 mL) and aqueous HCl (1.0 M, 5 mL) were added and phases were separated. The combined organic phases were washed with water (15 mL) and dried over anhydrous Na_2_SO_4_, filtered and evaporated to dryness. The crude phenol was purified by column chromatography on silica gel (eluent: EtOAc) to give **17** as orange crystalline solid (12 mg, 10%). M. p. 245–247 °C (dec.); ^1^H NMR (300 MHz, DMSO–*d_6_*): *δ* 11.50 (1H, br s), 7.95 (1H, s), 7.63 (1H, d, *J* = 12.3 Hz), 7.33 (1H, d, *J* = 1.5 Hz), 7.15 (1H, d, *J* = 2.7 Hz), 6.74 (1H, dd, *J* = 2.7 Hz, 12.3 Hz), 2.23 (3H, d, *J* = 1.5 Hz) ppm; ^13^C NMR (75 MHz, DMSO–*d_6_*): *δ* 186.3, 150.1, 148.3, 138.4, 137.4, 136.3, 134.3, 133.5, 131.1 (q, *J* = 5 Hz), 129.4, 128.0, 124.3, 123.4 (q, *J* = 271 Hz), 120.4 (q, *J* = 30 Hz), 12.3 ppm; HRMS-ESI *m/z*: calc. for C_15_H_9_F_3_O_2_ [M+H]^+^: 279.0633, found 279.0636.
